# An integrative modeling framework reveals plasticity of TGF-β signaling

**DOI:** 10.1186/1752-0509-8-30

**Published:** 2014-03-12

**Authors:** Geoffroy Andrieux, Michel Le Borgne, Nathalie Théret

**Affiliations:** 1 INSERM U1085, IRSET, Université de Rennes 1, 2 avenue Pr Léon Bernard, 35043 Rennes, France; 2 Université de Rennes 1, IRISA, 263 avenue du général Leclerc, 35042 Rennes, France

**Keywords:** TGF-β, Discrete dynamic model, Signaling pathways, Guarded transition

## Abstract

**Background:**

The TGF-β transforming growth factor is the most pleiotropic cytokine controlling a broad range of cellular responses that include proliferation, differentiation and apoptosis. The context-dependent multifunctional nature of TGF-β is associated with complex signaling pathways. Differential models describe the dynamics of the TGF-β canonical pathway, but modeling the non-canonical networks constitutes a major challenge. Here, we propose a qualitative approach to explore all TGF-β-dependent signaling pathways.

**Results:**

Using a new formalism, CADBIOM, which is based on guarded transitions and includes temporal parameters, we have built the first discrete model of TGF-β signaling networks by automatically integrating the 137 human signaling maps from the Pathway Interaction Database into a single unified dynamic model. Temporal property-checking analyses of 15934 trajectories that regulate 145 TGF-β target genes reveal the association of specific pathways with distinct biological processes. We identify 31 different combinations of TGF-β with other extracellular stimuli involved in non-canonical TGF-β pathways that regulate specific gene networks. Extensive analysis of gene expression data further demonstrates that genes sharing CADBIOM trajectories tend to be co-regulated.

**Conclusions:**

As applied here to TGF-β signaling, CADBIOM allows, for the first time, a full integration of highly complex signaling pathways into dynamic models that permit to explore cell responses to complex microenvironment stimuli.

## Background

Complex signaling by the polypeptide transforming growth factor TGF-β is one of the most intriguing networks that governs multifunctional processes and plays a pivotal role in tissue homeostasis and morphogenesis [1]. Whereas TGF-β is ubiquitously expressed in all cell types and tissues, its effects differ according to cellular type and microenvironment. For example, TGF-β inhibits epithelial cell growth but can promote stromal cell proliferation. The confounding pleiotropic effects of TGF-β derive from the complex nature of its signaling, whose full characterization requires modeling approaches that are still lacking. TGF-β signals through a heteromeric complex of two types of transmembrane serine/threonine kinases, the type I (T*β*RI) and type II (T*β*RII) receptors. TGF-β binding to T*β*RII induces the recruitment and phosphorylation of T*β*RI, which transduces signals to downstream intracellular substrates, the first of which are the R-Smad proteins. Once phosphorylated, R-Smad proteins hetero-dimerize with a common partner, CoSmad, and heterodimeric complexes move into the nucleus where they regulate the transcription of TGF-β target genes. Alternatively, non-Smad pathways involved in TGF-β signaling include several MAP kinase pathways, the Rho-like GTPase signaling pathways, and phosphatidylinositol-3-kinase/AKT pathways [2,3]. Hence, combinations of Smad and non-Smad pathways contribute to the high heterogeneity of cell responses to TGF-β. Moreover, the molecular actors of these pathways are part of other cell signaling networks activated by additional extracellular stimuli, leading to complex and extensive crosstalk that has been difficult to model.

Modeling the dynamics of the TGF-β canonical signaling pathway has followed classical differential approaches that either couple signaling together with receptor trafficking [4] or focus on Smad phosphorylation [5], Smad nucleocytoplasmic shuttling [6,7] and Smad oligodimerization [8]. In addition, integrative models have coupled receptor trafficking to Smad pathways [9-11]. We have taken advantage of these existing quantitative approaches to develop our own models, which we have applied to the study of the role of the ADAM12 tumor biomarker [12] and of the TIF1γ tumor suppressor [13]. These advances suggested that such small differential models could be developed into useful tools to investigate the role of new regulatory components of the canonical TGF-β signaling pathway. A major obstacle, however, is that differential equation-based models remain limited to a small number of reactions [14]. Because the explosion in the number of variables in complex networks makes parameterization intractable at the cellular scale [15], integration of the TGF-β signaling networks clearly requires other methods.

Alternative qualitative modeling approaches are based on discrete-event systems where each entity (genes or proteins) is represented by a finite-state variable and rules encode the possible states and interactions of biomolecules [16]. The two-state Boolean logic is the simplest discrete formalism and has been successfully applied for modeling specific signaling pathways such as those for the Epithelial Growth Factor Receptor [17] and insulin [18]. While Boolean approaches are based on switching functions and consider networks as logical circuits, other discrete formalisms such as rule-based methods allow for the biochemical and biophysical description of multi-state components [19]. For instance, agent-based modeling approaches have been employed to describe the behavior of TGF-β and EGF crosstalk in non-small-cell lung cancer models [20] and epithelial restitution [21]. Similarly, the role of TGF-β in epidermal wound healing has been deciphered using computational agent-based models [22]. More recently, hybrid models for tumor-stromal environment centered on TGF-β and EGF canonical pathways have described interactions between the extracellular matrix and growth-factor effects [23] and a related hybrid discrete-element cellular automata model has been proposed to understand how TGF-β modulates tumor-stroma interactions [24]. While informative, these studies remain partial and fail to account for the full complexity of TGF-β dependent signaling networks. A major challenge, then, is to integrate all available information within a dynamic model that fully addresses how TGF-β modulates heterogeneous cell responses.

The last decades have seen the accumulation of a rich trove of information about the molecular actors of signaling (extracellular stimuli, membrane receptors, transduction signal proteins, transcriptional factors) and their associated biochemical reactions (receptor activation, protein phosphorylation, cytoplasmic-nuclear shuttling, transcription). Considerable effort has gone into mining the literature to build signaling databases and provide a view of cell signaling pathways through descriptive graph-based representations (KEGG [25], Ingenuity Pathway [26], Biocarta, Reactome [27], Pathway Interaction Database (PID) [28]). While all of these approaches improve graph-based analyses of signaling pathways, the translation schemes used to translate biological data into discrete variables and the parameterization of reaction times relative to each other to obtain dynamic models raise many difficulties. Within a given database, distributed knowledge blends various biological concepts such as biochemical reactions and functional processes and includes heterogeneous levels of details that do not permit automatic translation into discrete models. More recent databases such as Reactome [27] and the Pathway Interaction Database (PID) [28] use a homogeneous concept of biological reactions based on mechanistic information to facilitate further formal interpretations and development of large-scale systems, and a Boolean framework was recently proposed for modeling cellular signaling from the whole Reactome database. In this case, however, parameterization of time remains to be achieved [29] as discrete models do not easily lend themselves to the handling of logical time based on partial ordering of events. In synchronous time models, all components change simultaneously while only one component of the state is allowed to change at each step of model evolution in asynchronous time models. While mixed-rule dating has been proposed to obtain a more realistic view of biological events [30,31], these approaches still fall short of successfully modeling signaling reactions.

To address these daunting difficulties, we have developed a new non-ambiguous formal interpretation of signaling pathways as discrete dynamic models. This interpretation differs from previous approaches in that it models the signal rather than the molecular biochemical network that transmits the signal. The resulting language, Computer-Aided Design for BIOlogical Models (CADBIOM), is based on a simplified version of guarded transitions [32] in which we introduced temporal parameters for each transition. This leads to high degree of flexibility in signal distribution and allows, for the first time, the construction of a fully integrative discrete dynamic model of TGF-β signaling pathways. To do so, we integrated the whole PID database into a single unified model containing 9177 biomolecules that include all TGF-β related signaling networks. Based on model-checking methods implemented in the CADBIOM application, we further analyzed all trajectories involved in the regulation of 145 TGF-β target genes included in the model. Importantly, clustering analyses of signaling trajectories successfully discriminate between Smad versus non-Smad-dependent genes and identify for the first time new combinations of extracellular stimuli involved in the regulation of TGF-β target genes. In addition, trajectory analyses are predictive for gene co-regulation as assessed by experimental gene expression data.

## Results and discussion

### Building a TGF-β CADBIOM model

To translate and integrate any kind of biological reaction involved in TGF-β signaling pathways, we developed a new language, CADBIOM, based on guarded-transition formalism (see Methods and Additional file 1: Figures S1 and S2). Briefly, a biological reaction is formalized as a transition from input biomolecules to output biomolecules under some conditions or guards. Conditions involve biomolecules as activators or inhibitors and the biological reaction takes place only if the inputs are present and the condition is true. We then consider that the occurrence of a biological reaction induces information propagation from each input to each output. Next, all the biological reactions are integrated by connecting the biomolecules: the target of a transition becomes the origin of the next one. The resulting network is turned into a dynamic system according to the choice of transitions that are fired at each step. To overcome the limitations of synchronous or asynchronous models, we introduce events, discrete signals that guide or restrain the choice of fireable transitions. The potential firing of reactions is dependent on the presence or absence of events that is formalized within the guard. By default, events are initially attributed to each transition, except for the formation or dissociation of complexes that share the same event since both components are simultaneously present.

**Figure 1:**
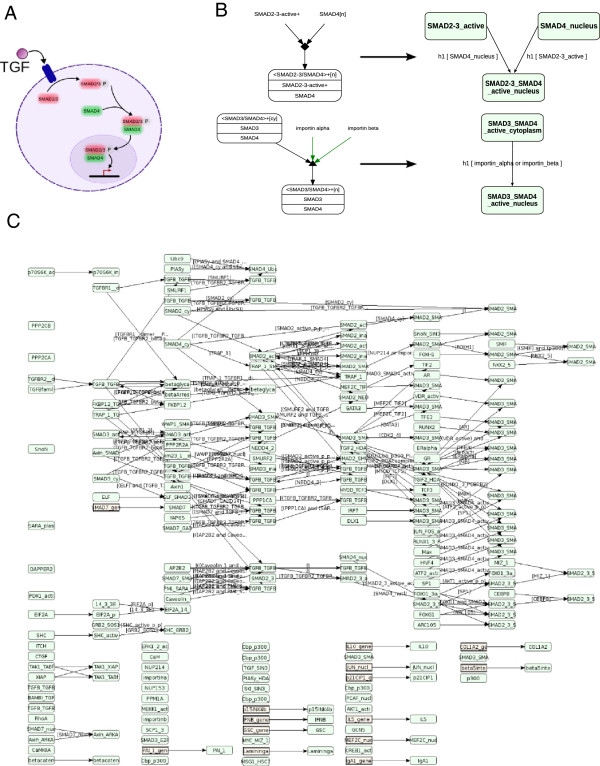
**CADBIOM model for the canonical TGF-β pathway. (A)** Schematic representation of the canonical pathway. Binding of TGF-β to the receptor induces recruitment and phosphorylation of Smad2/3. Activated Smad2/3 heterodimerizes with Smad4 and the complex migrates to nucleus where it regulates gene transcription. **(B)** Two examples of translations from the PID database (left) into the CADBIOM representation (right). Top: interaction of Smad2/3 with Smad4. The CADBIOM representation uses two transitions from a component toward the complex, conditioned by the presence of the other component and leading to the synchronization of the transition. The same event h1 is associated with the two transitions that occur simultaneously. Bottom: migration of the Smad complex to the nucleus. The CADBIOM representation uses a transition between the two places “SMAD3_SMAD4_active_cytoplasm” and “SMAD3_SMAD4_active_nucleus” conditioned by the presence of importins within the guard. The event h1 indicates the timing of the transition. **(C)** CADBIOM illustration of the canonical pathway after integration of the three PID pathways: “TGF-β receptor signaling” (PID Ref: tgfbrpathway), “regulation of cytoplasmic and nuclear Smad2/3 signaling” (PID Ref: Smad2-3pathway) and “regulation of nuclear Smad2/3 signaling” (PID Ref: Smad2-3nuclearpathway).

**Figure 2:**
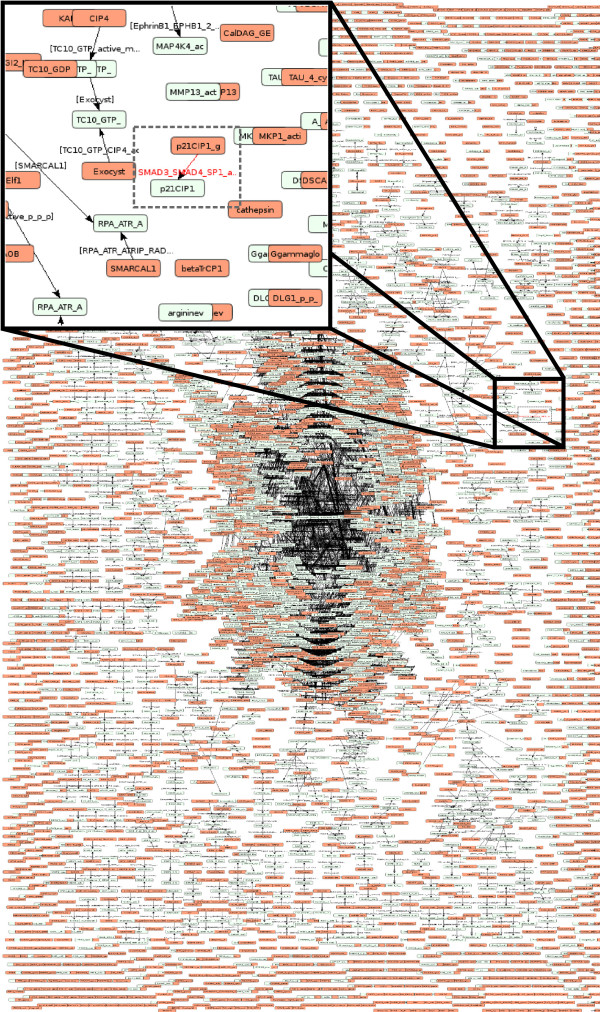
**A single unified model of the PID database.** The 137 pathways from the PID database containing 27876 biomolecules are integrated into a CADBIOM dynamic model containing 9177 places. Places without input are colored in orange. The insert illustrates an example of regulation by Smad3/Smad 4 and shows the transition of the p21CIP1 gene to p21CIP1 protein, which represents activation of the p21 gene. The condition for the transition contains an extensive logical formula that is not detailed here.

Taking advantage of the mechanistic view of the PID database, we extracted TGF-β signaling-pathway knowledge by developing a program that automatically translates XML files from PID into CADBIOM language. Three TGF-β signaling related pathways have been reported in PID: these include “TGF-β receptor signaling” (PID Ref: tgfbrpathway), “regulation of cytoplasmic and nuclear Smad2/3 signaling” (PID Ref: Smad2-3pathway) and “regulation of nuclear Smad2/3 signaling” (PID Ref: Smad2- 3nuclearpathway). According to the integrative rules described in Methods, we built the union of the three files by automatically removing redundancies (Figure 1). This model integrates 435 biomolecules and 141 reactions from PID, but only from the canonical Smad-dependent pathway. To extract all signaling information related to the non-Smad TGF-β signaling pathways, we had to incorporate information related to MAPK, Rho-like GTPase and phosphatidylinositol-3-kinase/AKT. A difficulty immediately arises in that these components are part of many other PID pathways; accordingly, all biomolecules that influence TGF-β signaling pathways need to be integrated. To overcome this obstacle and identify all components related to TGF-β, we first built a single CADBIOM model for the complete PID database. As illustrated in Figure 2, all 9248 reactions from 137 PID pathways were integrated into 9264 CADBIOM transitions. Note that transitions and reactions are not equivalent since a reaction for the formation of a complex between two biomolecules must be translated into two transitions. In contrast, two different reactions described in PID and sharing the same inputs and outputs can be translated into one or several CADBIOM transitions. The efficiency of integration is demonstrated by the reduction of the 27876 biomolecules that are part of the 137 PID pathways to 9177 non-redundant places in the CADBIOM model. Indeed, one PID biomolecule can be implicated in several reactions and/or pathways, while a CADBIOM biomolecule is represented by a unique place and all of its reactions are either incoming or outgoing transitions. It is important to note that the translation and integration of the 137 PID pathways into a single CADBIOM model does not alter the distribution of the ontology terms associated with biomolecules, ruling out loss of information resulting from integration (Additional file 1: Figure S3).

**Figure 3:**
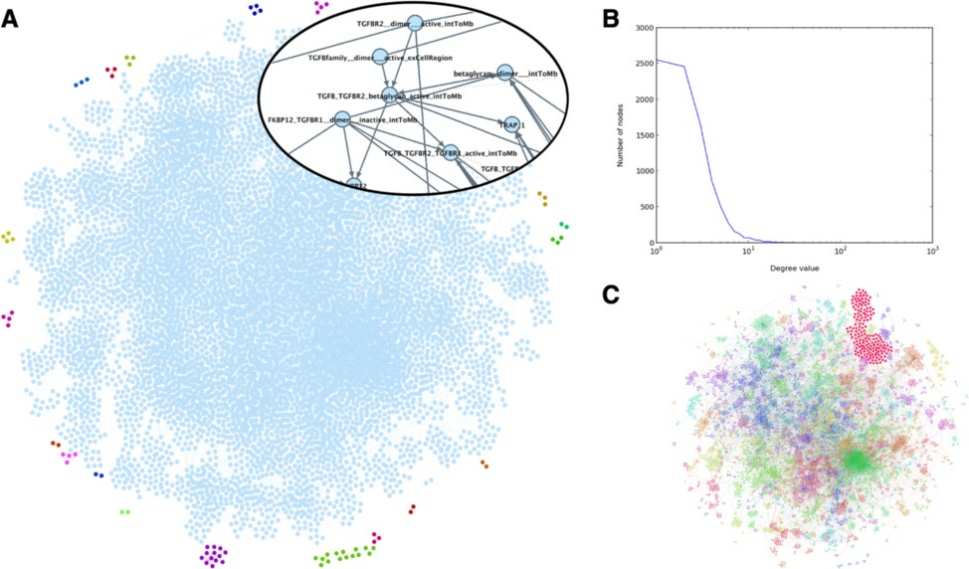
**Activation graph of the PID database. (A)** The activation graph is built using places from the CADBIOM model as nodes and the dependencies between places as edges. The dependency relationships include the transitions and the influence of conditions on the output of transition. The resulting activation graph contains 9077 nodes and 15499 edges. TGF-β belongs to the major connected component containing 8986 nodes (98% of nodes from the activation graph) demonstrating that all information from PID influences TGF-β signaling. Colors distinct from light blue denote non-connectivity of components. In the zoom view (insert), thin directed edges denote condition dependencies and thick directed edges transition dependencies. **(B)** Representation of degree distribution. **(C)** Modularity based hierarchical agglomerative clustering. Colors show the different clusters that are further detailed in Additional file 2: Table S1. A major specific module containing 168 places that include TGF-β, Smad proteins, TGF-β receptors and related molecules such as BMP (members of the TGF-β family) is shown as enlarged red nodes.

Based on this complete signaling model, we next sought to delineate parts of the network that influence TGF-β signaling pathways. We did this by creating an activation graph from all PID information using biomolecules as nodes and the dependencies between biomolecules as edges (Figure 3A). The dependency relationships include transitions and the influence of conditions on the output of transitions. The resulting influence graph illustrates all the relations between the biomolecules of the database, not only in terms of information propagation, but also in terms of regulation of reactions. Quite spectacularly, all the information from PID is highly connected with a major component containing 8986 nodes (98% of the total PID content) included TGF-β, indicating that integration of TGF-β signaling amounts to compiling the entire database. Most importantly, this integration is achieved without discarding non-canonical pathways that are not described as TGF-β-related in PID. Based on the distribution of the connectivity and modularity analysis, we showed that the network exhibits scale-free behavior (Figure 3B) and clustering of connected components identifies a TGF-β related associated module (Figure 3C, Additional file 2: Table S1). Taken together, our data provide the first dynamic discrete model of the TGF-β signaling network, including both Smad and non-Smad-dependent pathways, which integrates all networks that influence the cellular response to TGF-β. Because topological analyses of the activation graph could not be explain the heterogeneity of TGF-β signaling pathways, we further explored this highly complex model using model-checking approaches as described below.

### Application to the regulation of TGF-β dependent genes

Understanding how TGF-β induces heterogeneous biological responses is a critical question, which we addressed next by exploring the complexity of pathways that govern the regulation of TGF-β-dependent genes. Looking for signaling pathways that regulate a gene G coding for a protein P amounts to searching for all scenarios that verify the reachability of a property P (see Methods and Additional file 1: Figure S4). A scenario is a list of biomolecules activated at the initialization of the model. However, due to the size of the model and the wide set of potential solutions, we focused on minimal scenarios that take place in 10 steps, the representative size of solutions in our CADBIOM model. For each minimal scenario, we analyzed all possible trajectories to reach the property P, a trajectory being expressed as the list of biomolecules activated during signal propagation. Among the 679 genes described in PID, 145 have minimal scenarios containing the term TGF-β and correspond to extracellular stimulation by TGF-β.

**Figure 4:**
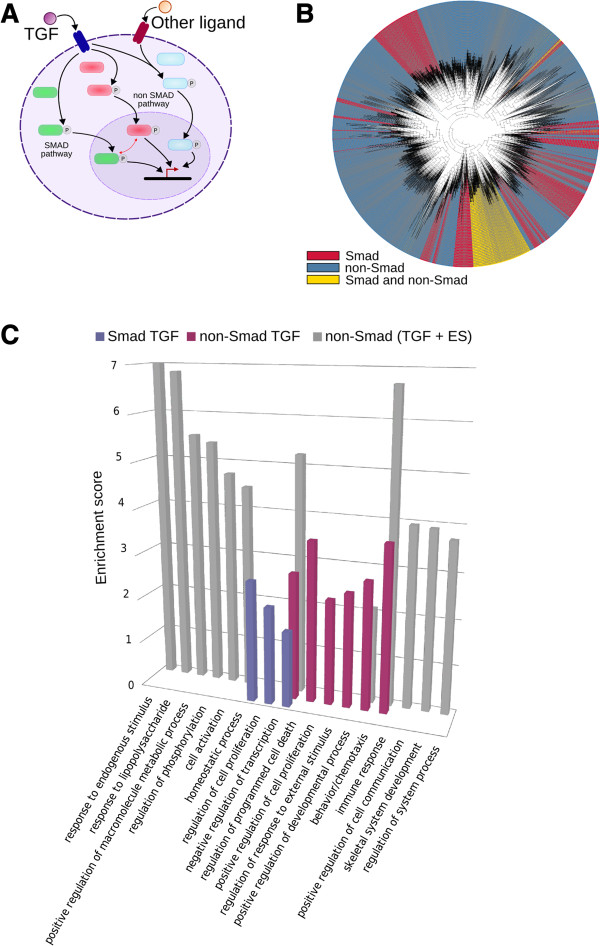
**Analysis of the 15934 signaling trajectories containing TGF-β. (A)** Schematic representation of the Smad and non-Smad TGF-β cell signaling pathways. **(B)** Trajectories are clustered according to their content in biomolecules using Euclidian distance metrics. Trajectories that contain Smad protein are colored in red, those that do not in blue and those containing both Smad and non-Smad proteins are in yellow. **(C)** Functional annotations of TGF-β dependent gene targets using the Database for Annotation, Visualization and Integrated Discovery (DAVID) tool. GOs are colored in grey, blue and red as indicated in the text. ES: extracellular stimuli.

To decipher the signaling regulatory network of TGF-β, we searched for all the signaling trajectories played according to these scenarios. We identified 15934 such trajectories. Based on their content in biomolecules, clustering analysis of trajectories showed that the presence or absence of the Smad intracellular signaling proteins defined specific groups (Figure 4 and Additional file 3: Table S2). As shown in Table 1, 18 TGF-β-dependent genes were reachable only if Smad proteins were present in their trajectories. An additional set of 5 genes was also found both with Smad- and non-Smad-dependent trajectories and clustered within a unique group, suggesting similar regulation. Four of these genes, DAPK1, COX_2/PTGS2, JUN and NOS2, are indeed functionally associated within the "cancer pathways" from the KEGG pathway database. CADBIOM also identified a much larger number of TGF-β-dependent genes that were reachable in the absence of Smad proteins, resulting in a set of 122 genes with non-Smad-dependent trajectories (See Additional file 4: Table S3). Importantly, all trajectories containing Smad proteins are associated with genes regulated by TGF-β as their unique extracellular stimulus, while trajectories induced by TGF-β associated with other extracellular stimuli never contained Smad proteins (Additional file 5: Table S4).

**Table 1:** Genes with SMAD-dependent trajectories identified by CADBIOM

**PID ID**	**HUGO ID**	**Description**
COX_2*	PTGS2	Prostaglandin-endoperoxide synthase 2
DAPK1 *	DAPK1	Death-associated protein kinase 1
GSC	GSC	Goosecoid homeobox
ID1	ID1	Inhibitor of DNA binding 1
IFNB	IFNB1	Interferon, beta 1
IgA1	IGHA1	Immunoglobulin heavy constant alpha 1
IL10	IL10	Interleukin 10
IL5*	IL5	Interleukin 5 (colony-stimulating factor, eosinophil)
JUN*	JUN	Jun oncogene
KLK2	KLK2	Kallikrein-related peptidase 2
Laminin gamma1	LAMC1	Laminin, gamma 1
MEF2C	MEF2C	Myocyte enhancer factor 2C
NOS2*	NOS2	Nitric oxide synthase 2, inducible
p15INK4b	CDKN2B	Cyclin-dependent kinase inhibitor 2B (p15, inhibits CDK4)
p21CIP1	CDKN1A	Cyclin-dependent kinase inhibitor 1A (p21, Cip1)
RAB7	RAB7A	RAB7A, member RAS oncogene family
SMAD7	SMAD7	SMAD family member 7
SNAI1	SNAI1	Snail homolog 1 (Drosophila)
SOCS3	SOCS3	Suppressor of cytokine signaling 3
TCF3	TCF3	Transcription factor 3
TLX2	TLX2	T-cell leukemia homeobox 2
TRPC6	TRPC6	Transient receptor potential cation channel, subfamily C, member 6
TRPV1	TRPV1	Transient receptor potential cation channel, subfamily V, member 1

The Table lists genes with SMAD-dependent trajectories. Gene names (ID) are given according to their nomenclature in the PID and HUGO databases, respectively, and are followed by their description. Asterisks denote genes that are also found with non-SMAD-dependent trajectories (see Text and Additional file 4: Table S3).

To assess the functional association of target genes within each group, we performed gene ontology annotation using the Database for Annotation, Visualization and Integrated Discovery, DAVID (Figure 4C). Remarkably, genes regulated by the Smad-dependent TGF-β pathway were found to be specifically associated with the biological processes that include the GO terms “regulation of cell proliferation”, “negative regulation of gene expression” and “regulation of programmed cell death” (blue color). In contrast, genes regulated by non-Smad-dependent pathways (grey or red) were always associated with multiple biological processes according to the absence or presence of other extracellular stimuli. The non-Smad pathways (colored in red) containing only TGF-β were found to regulate genes with GO terms “immune response and response to external stimulus”, “positive regulation of cell proliferation” and “developmental process, behavior and regulation of programmed cell death”. In cases where non-Smad pathways linked TGF-β to other stimuli in their trajectories, we observed gene expression responses that were part of Smad-dependent TGF-β trajectories (*e.g.* “programmed cell death” GO) or of non-Smad-dependent trajectories (*e.g.* “immune response” GO), as well as a broader range of responses belonging to GOs such as “metabolism”, “development” and “homeostasis”.

**Figure 5:**
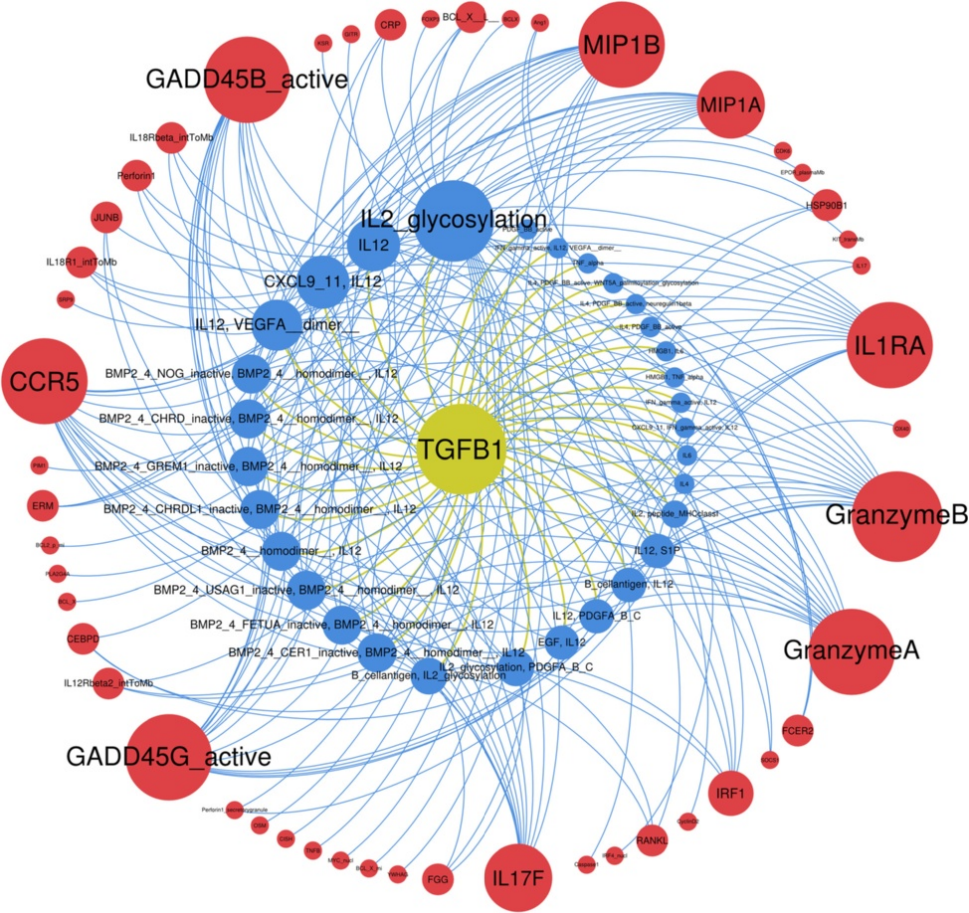
**Plasticity of gene responses to combinations of TGF-β with other extracellular stimuli.** 49 genes (red nodes) are regulated through signaling trajectories containing TGF-β (yellow node) and at least one other extracellular stimulus (blue nodes). The size of the nodes is relative to the number of edges. CCR5, GADD45A/B, MIP1A/B, GranzymeA/B, IL1RA and IL17F are the most represented genes and share the largest number of combinations in their trajectories.

Altogether, we identified 31 combinations where TGF-β was linked to other extracellular stimuli, illustrating the high degree of plasticity of TGF-β gene regulation (Additional file 5: Table S4 and Figure 5). Among these combinations, 18 associate TGF-β with IL12 and are involved in the regulation of 9 genes: CCR5, GADD45A and GADD45B, MIP1A and MIP1B, Granzyme A and Granzyme B and IL17F and IL1RA. Interestingly all these genes are functionally linked to viral infection/inflammation and stress response, suggesting that different combinations of stimuli can lead to a similar biological function. Indeed CCR5 is a beta-chemokine receptor that binds HIV, and Mip1A and B are major HIV-suppressive factors that bind CCR5. Additionally, Granzyme A and B are serine proteases that mediate apoptosis of virus-infected cells and IL1RA and IL17F act as proinflammatory cytokines. Finally, GADD45A/B are transcriptional factors that mediate global response to environmental stress. These functional links revealed by CADBIOM analysis have not been previously reported using other modeling approaches. Taken together, these data are in accordance with and strengthen the known concept of Smad- and non-Smad-dependent TGF-β pathways and provide for the first time trajectories for regulatory ligands. Of note also is the identification, through CADBIOM analyses of trajectories for gene regulation, of the 31 combinations that associate TGF-β with other extracellular stimuli to drive gene regulation through Smad-independent pathways. In addition, CADBIOM identifies the TGF-β/IL12 association as a novel basic module for signaling networks involved in the regulation of 9 genes implicated in the response to viral infection. Because such complex associations cannot be evaluated by classical experimental approaches using *in vitro* cell stimulation, we investigated the biological relevance of these combinations by analyzing co-expression of genes sharing extracellular stimuli.

### CADBIOM trajectories are associated with co-expression of genes

**Figure 6:**
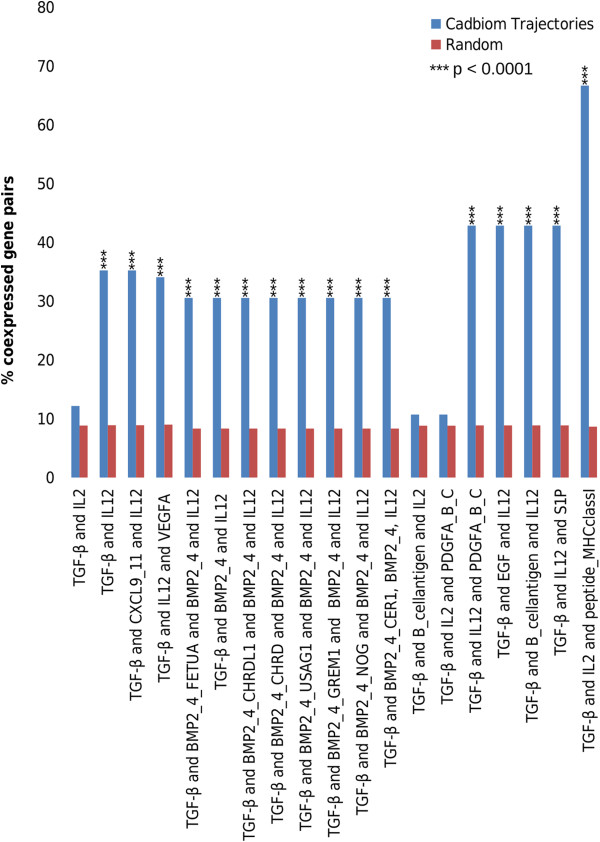
**Association between extracellular stimuli involving TGF-β and gene co-regulation.** For each gene pairs sharing combinations of extracellular stimuli (blue), we extracted the experimental data of gene co-expression from the Gemma database. Results are expressed as the ratio of co-expressed genes to possible gene pairs. Controls (red) are random gene pairs (n = 1000) from Gemma.

**Figure 7:**
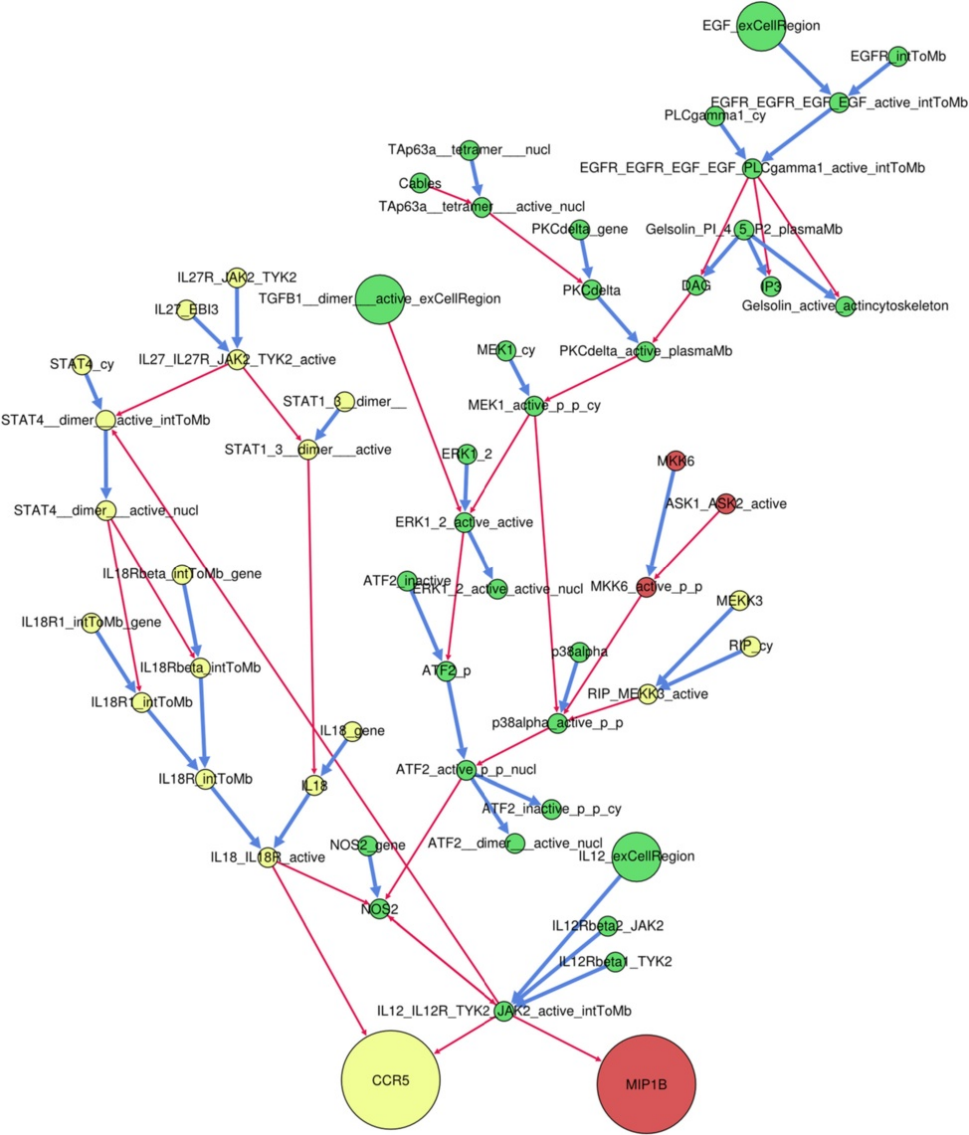
**Common-trajectory network for two co-regulated genes, MIP1B and CCR5.** Solutions for reachability of MIP1B and CCR5 genes were calculated using the CADBIOM application and trajectories for each solution were compiled in a graph representation. Blue arrows symbolize transitions and red arrows conditions. Yellow nodes are specific to CCR5 trajectories, red nodes are specific to MIP1B trajectories and green nodes are common to both CCR5 and MIP1B trajectories.

The cellular microenvironment induces numerous simultaneous stimuli and cells respond according to the integration of all of these signals. For a given set of stimuli, signaling pathways activate gene expression profiles that can be identified using transcriptomic methods. Based on this supposition, we expected that two genes regulated by TGF-β together with other extracellular stimuli might be found to be co-regulated in biological samples. To test this hypothesis, we analyzed gene expression of each pair of genes sharing extracellular stimuli by using experimental data from Gemma, a database for the meta-analysis of gene expression profiles [33]. Based on the available set of 2110 Gemma human profiling expression studies, we determined whether two genes sharing a combination of TGF-β and another stimulus are co-expressed or not. In this case, 19 out of identified 31 combinations were found to regulate at least two genes. As shown in Figure 6, gene pairs activated by similar combinations were found to be significantly co-expressed, validating the biological relevance of trajectory analyses. As illustrated in Figure 7 for CCR5 and MIP1B, CADBIOM analysis can lead to the identification of genes that are co-expressed and share combinations, thus uncovering new and highly complex common trajectory networks.

To generalize this key observation to any genes from the model, we randomly generated 649 pairs of genes present in both the CADBIOM model and the Gemma database (Additional file 6: Table S5). Extracting all gene pairs that share signaling trajectories from the CADBIOM model and the corresponding co-expression data from Gemma reveals that 81% of co-expressed gene pairs in Gemma share regulatory signaling trajectories in the CADBIOM model. In contrast, 82% of non co-expressed gene pairs did not share regulatory pathways. These results demonstrate that trajectories identified by CADBIOM are highly predictive for gene co-expression in biological samples.

In summary, CADBIOM constitutes a new formalism for modeling signaling networks and provides the first discrete dynamic model for TGF-β signaling pathways. Based on model-checking methods, we describe the highly complex signaling trajectories that regulate TGF-β-dependent gene targets and we demonstrate that combinations of TGF-β with other extracellular stimuli lead to non-Smad-dependent pathways. Finally, we show that CADBIOM modeling allows for the identification of complex trajectories induced by multiple extracellular stimuli, successfully predicting gene co-expression patterns and biological functions that cannot be characterized by direct experimental approaches. More generally, we expect that CADBIOM will be of broad use to help unravel other highly complex pathways that have proven intractable using classical modeling approaches. To provide for detailed explorations of signaling models, CADBIOM design and analysis, all required tools have been implemented into a user-friendly open-source application package that is readily available to researchers (http://cadbiom.genouest.org).

## Conclusions

Signaling pathway networks orchestrate cell life through a uniquely connected complex molecular circuitry for propagation of information. Because of the huge number of biological reactions that are implicated, modeling cell signaling to predict cell responses to this huge flow of information is a major challenge that requires new approaches if all information is to be dynamically integrated. In the present work, we have developed a new formalism, CADBIOM, based on guarded transitions and combined discrete abstraction to propose, for the first time, a fully integrated model for TGF-β signaling pathways.

Major issues in modeling biological large-scale phenomena are the collection of information from the literature. While cell signaling pathways are described in numerous databases, a recent report demonstrated a high degree of inconsistencies when different databases are compared [34]. Based on the Jaccard similarity coefficient, the authors compared four well understood pathways that involve the cytokines EGF (Epidermal growth factor), TGF-β (Transforming growth factor), TNFα (Tumor necrosis factor) and the signaling protein WNT (wingless-type) described in six databases, including GeneGo (http://www.genego.com), KEGG [25], NCI-PID [28], NetPath [35], PANTHER [36] and Reactome [27]. Only 10% similarity was found, suggesting that the description of each pathway is database- or even curator-specific. To circumvent such potential pitfalls, we extracted instead all relevant information from TGF-β pathways using a single database, PID, integrating them into a single unified model. The choice of PID is fully justified by the observation that, unlike other databases, PID formalizes signal propagation instead of describing biochemical reactions. This means that an interaction is described as a biological event that includes its participating molecules and conditions, an interaction which consumes its inputs and produces its outputs. This overall description is close to that used for guarded-transition systems [32], a state/event formalism that can represent both flow circulation with transitions and natural composition rules, and remote influences with transition guards. The direct translation of the complete XML-formatted database content into CADBIOM formalism then allows for the integration of information and automatically creates the first dynamic model of the TGF-β signaling network.

Three TGF-β signaling pathways are described in PID: TGF-β receptor signaling, regulation of cytoplasmic and nuclear Smad2/3 signaling and regulation of nuclear Smad2/3 signaling (see Figure 1) and summarize the canonical TGF-β pathway. We compiled these into CADBIOM and, importantly, also included non-Smad TGF-β pathways and all their influencing components (see Figure 2). The resulting single unified CADBIOM model is a highly connected graph containing 9177 places where 98% of nodes directly or indirectly influence TGF-β-dependent signals. Note that this high connected component contained also other receptor/factors. An important point to consider in automatic translation from a database is the continuous upgrading of the generated model. PID upgrading was recently stopped and closing of the PID project, a collaborative effort between the National Cancer Institute (NCI, Besthesda) and the Nature Publishing Group, has been announced. Fortunately, the Reactome project from the EBI consortium shares information with PID, which already imported biological data from Reactome’s BioPAX2. While some limitations in the specifications of Reactome’s BioPAX2 were previously reported by PID’s authors [28], the recent upgrade of BioPAX 2 to BioPAX3 [37] should facilitate data extraction from Reactome into CADBIOM.

Besides the straightforward structural analyses of large integrated graphs, the critical point in building signaling models is to describe the dynamics of signal propagation. We have accommodated this key requirement by using a state/event formalism based on guarded transitions that differ from traditional ones through the use of event algebra. Formalisms used in UML (Unified Modeling Language) or in state-charts [38] include an event in the transition guard but lack an operation for combining events into new events. In contrast, using CADBIOM formalism, the timing of model evolution is directly linked to the biological reactions by associating an event to each reaction. Consequently and although the transition systems use a Boolean expression for the guard, CADBIOM formalism differ from classical Boolean approaches [29,39-42] because the temporal order of the reaction is not forced (synchronous or asynchronous) but included in the guard. CADBIOM modeling permits simultaneous changes to several reactions and the exploration of all potential behaviors. The resulting modeling framework is much richer since simultaneous reactions are neither excluded nor imposed.

Biological models mainly focus on the search for steady states [43]. However, while such approaches make sense for gene regulatory or metabolic networks, they are not appropriate for modeling signal propagation. CADBIOM overcomes this limitation by implementing tools based on model-checking methods to investigate the reachability of properties instead [44,45]. These analyses usually require the computation of highly complex state-transition diagrams, which contain approximately 2^9000^ (10^2709^) states in our model. To avoid graph calculus, we use propositional logic and SAT solver-based approaches that have been demonstrated to be efficient for characterizing biochemical networks [46]. We have used these approaches to analyze scenarios and trajectories involved in the activation of the 145 TGF-β-dependent gene targets identified by our unified model. We demonstrate that scenarios can indeed discriminate between Smad and non-Smad-dependent pathways and that TGF-β associates with other extracellular stimuli to regulate non-Smad-dependent genes that are functionally related to shared biological processes. In these cases, the complexity of functions regulated by non-Smad pathways is mainly due to the association of TGF-β with other extracellular stimuli. We note that such a context-dependent role for external stimuli has been previously suggested by a global analysis of the effects of pair-wise ligand combinations [47]. However, these experimental approaches strongly limit the number of combinations that can be studied and a broad analysis of signaling trajectories is impossible. Using discrete models and model-checking approaches, we reasoned in the opposite way and, instead of performing TGF-β stimulation to identify signaling toward gene targets, we investigated trajectories starting from genes, thereby allowing identification of complex extracellular stimuli that include TGF-β.

Classical modeling approaches perform simulations using extracellular stimuli or activation of receptors at the initialization step to mimic experimental conditions in cell culture. In contrast, CADBIOM uses reachability properties to explore all signaling pathways without *a priori* on external stimuli that may be combined with TGF-β. This constitutes a radically different approach that is closer to the biological reality of cells, which live in a complex environment where TGF-β is always a part of stimuli. As a case in point, our study identifies a novel TGF-β-dependent network containing 9 viral response-related genes that are regulated by combinations that chiefly involve IL12, a cytokine with diverse functional effects [48]. The anti-inflammatory effect of TGF-β has been proposed to modulate the functions of IL12 in the immune response [49,50], but pathological contexts involving changes in environmental stimuli lead to a pro-inflammatory role for TGF-β [51,52]. These observations illustrate the need to investigate complex TGF-β models that include all potential influences and take into account all physio-pathological contexts. CADBIOM provides for such integration by creating a new state-event formalism to integrate biological reactions into a dynamic model for signal propagation. Finally, the creation of the first single unified model of the TGF-β signaling network, which integrates both Smad- and non-Smad-dependent pathways, constitutes an important landmark and provides a unique and powerful tool for the full exploration of TGF-β-dependent functions. More generally, the new computational approach provided by CADBIOM for modeling signaling networks should improve our overall understanding of cellular responses to complex stimuli, as illustrated here by the extraordinarily complex example of TGF-β signaling.

## Methods

### CADBIOM

CADBIOM software is covered by a GNU-public license that permits the conception, simulation and questioning of the discrete dynamic models describe in this article. All these features can be reached using the graphical user interface or through its application programming interface (API). Automatic conception of models is made possible through the PID database translation scheme (XML format). Resulting models can be stored in Cadlang, a text representation of CADBIOM models. CADBIOM is freely available at http://cadbiom.genouest.org/.

### Model formalism

CADBIOM formalism is based on guarded transitions and the introduction of discrete signals called events. A guarded transition is graphically represented by: A ^h[Cond]^→ B. A and B are respectively the origin and target places of the transition. The guard of the transition is composed by an event h and a condition Cond, a logical formula with places as variables and ∨, ∧ and ¬ as logical operators.

Places are state variables that take Boolean values and represent biomolecules such as proteins or complexes. An event is the mathematical concept that denotes an occasional occurrence. An event has a name h and a singleton domain to denote the occurrence of the event. At each step, an event can occur (denoted by the symbol T) or not (denoted by the symbol **⊥**). A realization of a finite set of events (h_i_)_i∈*I*_ is a sequence of elements of {T, **⊥**}^*I*^ \{ **⊥**^*I*^ }. To handle integrated models containing large amount of data, we combine events and states. Operations on events are well known in computer science. The two basic operators are a merge that corresponds to multiplexing and a selection of occurrences corresponds to under-sampling. CADBIOM borrows the default operator and the when operator from the Signal language [53]. The default operator merges the two events *h*_1_ and *h*_2_. The event *h* = (*h*_1_ default *h*_2_) is present when either the event *h*_1_ or the event *h*_2_ is present, h is absent otherwise. The when operator is an operator between events and logical combinations of state variables and selects occurrences of an event when the propositional formula evaluates to True on its right-hand side. The event *h* = (*h*_1_ when B) is present when *h*_1_ is present and B is True, it is absent otherwise.

### Model simulation

The system evolves according to transition firings that change the value of places, which is either *True* or *False* at any step. Given a guarded transition A ^h[C]^_→_ B, we define the transition event as *h*_tr_ = *h* when (A ∧C). The transition event *h*_tr_ is present if and only if h is present and (A ∧ C) is *True*, that is, when the input place is *True* and the condition C is verified. When a transition is fired, the source is inactivated and the target is activated. The evolution function of a state A relies on the current value A_k+1_ at step k to the next value A_k+1_ at step k + 1.

The state of a place changes if either an in-transition or an out-transition is fired. In the case of simultaneous firing, we postulate that activation prevails over inactivation.

We define two events associated with a place A by:

$$h_{\mathrm{in}}=\mathrm{default}\;t_{r}\in T_{\mathrm{in}}\;h_{\mathrm{tr}}$$

$$h_{\mathrm{out}}=\mathrm{default}\;t_{r}\in T_{\mathrm{out}}\;h_{\mathrm{tr}}$$

The *h*_in_ event is present if at least one of the in-transitions is fired. The *h*_out_ event is present if at least one of the out-transitions is fired. The mathematical formalization of the rules is given by:

$$A’=\left(h_{\mathrm{in}}\;\mathrm{default}\;\neg h_{\mathrm{out}}\right)\;\mathrm{default}\;A$$

where *A*’ is the value of A at next step.

The initial condition for simulation analyses requires the design of activated places (called scenario) and a specific timing. CADBIOM proposes either a manual selection or an automatic selection of “frontier” places. Frontier is the set of places which cannot be activated from inside the model. In guarded transition models, places without input transitions belong to the frontier. The script for events timing is provided during reachability analyses as a txt file that can be loaded in simulation tool

### Property search

The temporal properties of models are explored using methods based on model checking. The aim of these methods is to determine an allocation of state variables and event timing that verify a property, that is, any logical formula composed of model places and logical operators ∨, ∧ and ¬. To investigate the reachability of biological properties such as "how to express a given gene?", we search for the allocation of states variables and event timing that lead to the activation of the place symbolizing the gene. We focus on frontier places because signaling networks are usually polarized from the extracellular environment to the nucleus. A frontier is the set of places without any in-transitions that cannot be activated from inside the model. All other places are initialized in an inactivated state.

We first generated the formulas that describe the evolution of the system. Using the evolution rules above, dynamic models are translated into propositional logic formulas using the Tseitin translation. The resulting formulas are under conjunction normal form (CNF) and qualify the dynamics between steps *i* and *i* + 1. We then unfold the trajectory from step 0 to n. The whole formula is then given to a SAT solver to find an allocation of state variable and event timing that satisfies the formula.

We next define a scenario as the (F; T ) pair, where F is a set of places which are activated at the initialization step and T is a sequence of sets of events *h*. This reachability property is then used to search for minimal scenarios such that the property is not achieved as soon as one component is removed in the initialization places or as soon as a component of event timing is disabled. A scenario is said to be minimal if, for any place *A* ∈ F, (F \ {A}, T ) is not an activation condition and for any *i* < *n* and any *h* ∈ *H*_i_, (F; (*H*_1,….,_*H*_*i*_ \{h}, …,*H*_n-1_)) is not an activation condition. Any scenarios can be simulated to retrieve all the activated places that lead to reachability of the property. These activated places compose a trajectory.

### Compilers

CADBIOM software includes several compilers sharing the same back-end. The front-ends compile PID XML files, CADBIOM XML files and Cadlang files into an intermediate representation of guarded-transition models. The common back-end generates logical constraints in propositional clause form. During this step, constant propagation and common sub-expression elimination optimizations are performed. Combined with many peephole optimizations, these techniques allow the back-end to generate reduced sets of constraints that facilitate the work of the SAT solver.

### Resources

The CADBIOM model is a translation of the PID database content (http://pid.nci.nih.gov/). See Additional file 1: Figure S1 for the complete translation scheme. Model-checking analysis is performed using the cryptominisat SAT solver (http://www.msoos.org/cryptominisat2/). Graph representations of solutions are displayed using Gephi, an open-source platform for graph visualization (https://gephi.org/).

## Additional files

**Additional Material.** The following additional data are available with the online version of this paper. Additional data file 1 is a figure describing the scheme for translating biological reactions from PID database into CADBIOM formalism. Additional data file 2 is a figure illustrating translation schemes for the TGF-β model. Additional data file 3 is a figure showing the conservation of ontology during the translation process. Additional data file 4 is a figure detailing the procedure for calculating reachability. Additional data file 5 is a text detailing mathematicals semantics for CADBIOM formalism.

## Competing interests

The authors declare that they have no conflict of interest.

## Authors' contributions

GA conceived the study, developed the software and drafted the manuscript. MLB conceived the study, developed the software and drafted the manuscript. NT conceived the study and drafted the manuscript. All authors have read and approved the final manuscript.

## Supplementary Material

Additional file 1: Figure S1 Translation scheme for biological reactions into guarded transitions. **Figure S2.** Illustration of translation schemes for the TGF-b model. **Figure S3.** Conservation of biological annotations. **Figure S4.** Reachability of a property P. **Supplementary Methods.** mathematical semantics. Click here for file

Additional file 2: Table S1 Modularity classes in activation graph. The activation graph has a significant modularity score of 0,819 that reveals a non-random distribution of edges and a high connectivity between nodes inside classes. Most of TGF-β signaling-related components, including TGFβ, TGFβR and SMAD proteins belong to modularity class 10 that contains also TGF-β superfamily members like BMP. Click here for file

Additional file 3: Table S2 Vector matrix of trajectories that regulate BCL_X_L, JUNB, CRP, FGG, IRF1, CEBPD, RANKL genes. The occurrence of components is expressed as 0 (absent) and 1 (present) in trajectories. Click here for file

Additional file 4: Table S3 Genes with non_Smad-independent trajectories identified by CADBIOM. The Table lists genes with non-Smad-dependent trajectories. Gene names (ID) are given according to their nomenclature in the HUGO and PID databases, respectively, and are followed by their description. Click here for file

Additional file 5: Table S4 Target genes regulated by combinations of extracellular stimuli identified by CADBIOM. Proteins that serve as extracellular stimuli and their combinations thereof are listed together with their target genes, listed according to the PID nomenclature. Click here for file

Additional file 6: Table S5 List of the 649 pairs of genes randomly chosen to evaluate the association between co-expression and trajectories. Click here for file
