# Supplementary material for: An integrative modeling framework reveals plasticity of TGF-β signaling
Source: BMC Syst Biol. 2014 Mar 12;8:30. doi: 10.1186/1752-0509-8-30 (PMC4007780; doi:10.1186/1752-0509-8-30)
Supplement: Additional file 1: Figure S1 — Translation scheme for biological reactions into guarded transitions. Figure S2. Illustration of translation schemes for the TGF-b model. Figure S3. Conservation of biological annotations. Figure S4. Reachability of a property P. Supplementary Methods. mathematical semantics. [file 1752-0509-8-30-S1.pdf]

Additional material

Geoffroy Andrieux, Michel Le Borgne and Nathalie Théret

Table of contents for Additional material:

Page 2 Supplementary Figure 1: Translation scheme for biological reactions into guarded transitions.
Page 5 Supplementary Figure 2: Illustration of translation schemes for the TGF-β model.
Page 7 Supplementary Figure 3: Conservation of biological annotations.
Page 8 Supplementary Figure 4: Reachability of a property P.
Page 9 Supplementary Methods: mathematical semantics

Supplementary Figure 1: Translation scheme for biological reactions into guarded transitions.

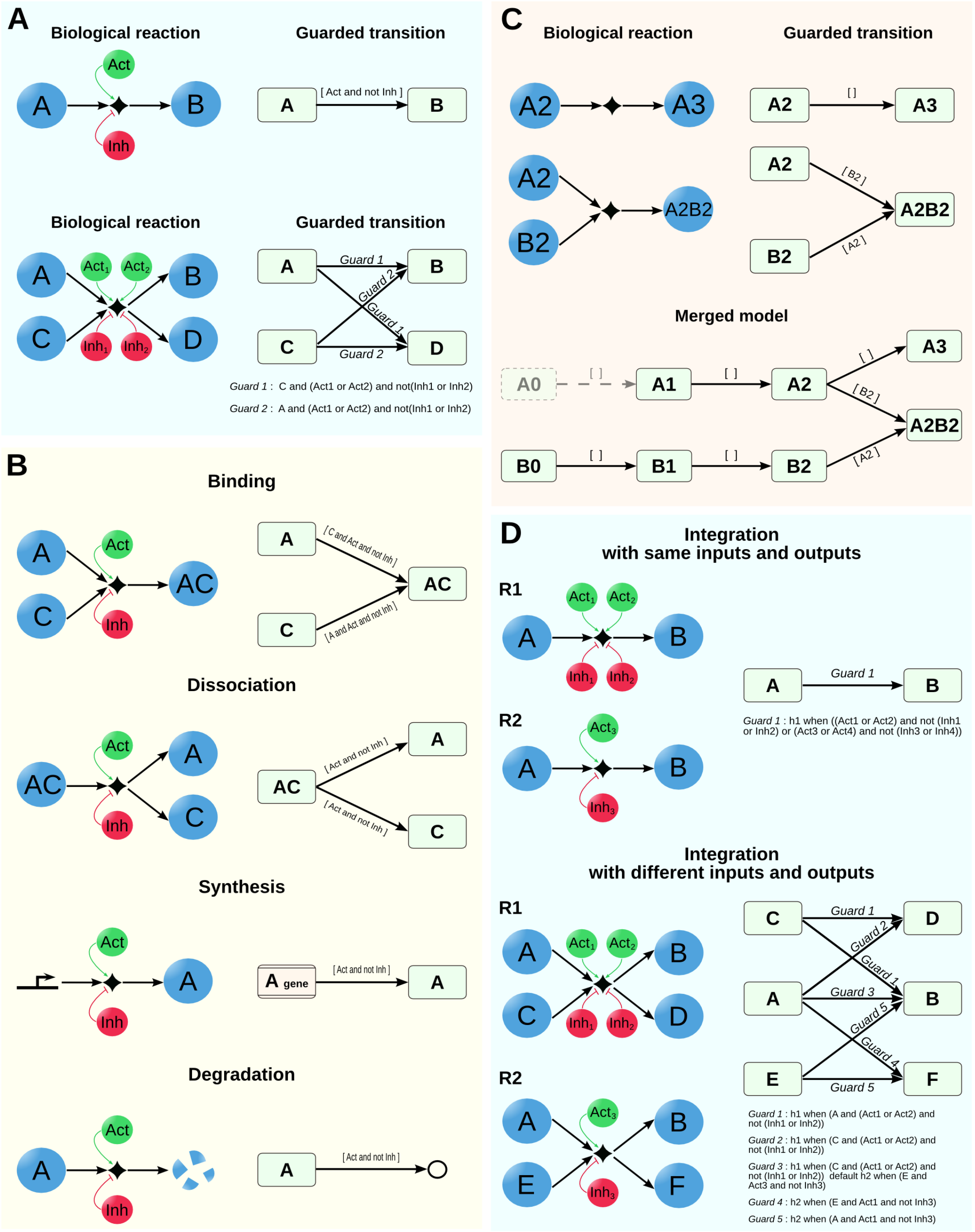

Supplementary Figure 1: Translation scheme for biological reactions into guarded transitions.
(**A**) Top: General case of a biological reaction where biomolecule A gives rise to biomolecule B. The regulation of the reaction is symbolized by activators Act and inhibitors Inh. The interpretation into a guarded transition (top right) from place A to place B is symbolized by rounded rectangles; the Act and Inh regulators form the guard of the transition. Bottom: generalization to a biological reaction with several inputs and outputs. Because all the inputs are required for the reaction to occur, the input C appears in the $Guard1$ of the transition $A \longrightarrow B$ and A appears in the $Guard2$ of the transition $C \longrightarrow B$. In addition, the inputs must be combined with other conditions within the guards. Combining inhibitors and activators in a transition guard requires detailed biological information. The combinatorics of inhibitors/activators are rarely well documented, if at all, in the literature. In the absence of information, we can combine activators and inhibitors either with the conjunction "and" or the disjunction "or". Using the "or" option, the $Guard1$ combines the input C with activators and inhibitors as follows: $C\ and\ (act1\ or\ act2)\ and\ not\ (Inh1\ or\ Inh2)$.

(**B**) Translation scheme for different categories of biological reactions into guarded transitions. To represent protein synthesis, we introduce a permanent place (double-lined rectangle) that symbolizes the corresponding coding gene and which, unlike other places, is never inactivated by an outgoing transition. On the contrary, trap places (black circle) are introduced for degradation processes and do not have outgoing transitions.

(**C**) Representation of the specific case where two reactions share the same biomolecule $A2$ (top). Merging of the reactions (bottom) leads to competition and the data-flow semantics do not allow the activation of place $A2B2$. Difficulties arise from reactions that share the same input and/or output biomolecules. $A2$ is involved in two reactions and can either be transformed into $A3$ or make a complex with $B2$. In this case, the transition $A2 \xrightarrow{[\,]} A3$ is fired when $A2$ alone is activated while the transition $A2 \xrightarrow{[B2]} A2B2$ is fired when $A2$ and $B2$ are both activated. Initializing the model with $A1$ and $B0$, and using the data-flow evolution rule, the transition $A2 \xrightarrow{[B2]} A2B2$ does not occur as it formally requires two transitions, ($B0 \xrightarrow{[\,]} B1$, $B1 \xrightarrow{[\,]} B2$), whereas only one transition, ($A1 \xrightarrow{[\,]} A2$), is required to render $A2 \xrightarrow{[\,]} A3$ fireable. In more simple terms, according to the data-flow scheme, $A2$ is consumed before $B2$ is available. Adding a transition $A0 \xrightarrow{[\,]} A1$ (dotted line) allows for the formation of the $A2B2$ complex if the model has been initialized with $A0$ activated instead of $A1$. Such a postulate illustrates how the schedule of the model depends on the number and configuration of the transitions. To overcome such limits, we introduce events to allow for delays in transition firing, as indicated in Materials and Methods. One way to fire the transition $A2 \xrightarrow{[B2]} A2B2$ is to modify the timing by adding an event h in each transition guard as follows: $B0 \xrightarrow{h_1[\,]} B1, B1 \xrightarrow{h_2[\,]} B2, B2 \xrightarrow{h_3[A2]} A2B2, A2 \xrightarrow{h_3[B2]} A2B2, A1 \xrightarrow{h_4[\,]} A2$, $A2 \xrightarrow{h_5[\,]} A3$. We use the same event h_3 for both $A2 \xrightarrow{h_3[B2]} A2B2$ and $B2 \xrightarrow{h_3[A2]} A2B2$ transitions since complex formation implies simultaneous firing of the two transitions. The introduction of events permits the activation of $A2B2$ when $A1$ and $B0$ are activated at the initial step if the following realization of events is enforced:

$Steps$	1	2	3
h_1 :	$\top$	$\bot$	$\bot$
h_2 :	$\bot$	$\top$	$\bot$
h_3 :	$\bot$	$\bot$	$\top$
h_4 :	$\top$	$\bot$	$\bot$
h_5 :	$\bot$	$\bot$	$\bot$

Using this schedule, the following transitions are fired and $A2B2$ is activated:

$$\begin{array}{cccccc} & 1 & & 2 & & 3 \\ A1 & \xrightarrow{h_4[\,]} A2 & B1 & \xrightarrow{h_2[\,]} B2 & A2 & \xrightarrow{h_3[B2]} A2B2 \\ B0 & \xrightarrow{h_1[\,]} B1 & & & B2 & \xrightarrow{h_3[A2]} A2B2 \end{array}$$

(**D**) Representation of the integration of reactions that share the same biomolecules. Top: To integrate $R1$ and $R2$ biological reactions that share the same inputs and outputs but have different regulators (inhibitors and activators), we generate two conditions: $cond1 = (Act1\ or\ Act2)\ and\ notInh1\ or\ Inh2)$ for $R1$ and $cond2 = (Act3\ and\ not\ Inh3)$ for $R2$. The new condition for the transition $A \rightarrow B$ resulting from the two reactions is then $cond1\ or\ cond2$. The event of the transition is a variable $h1$ since an occurrence of $R1$ cannot be distinguished from an occurrence of $R2$. Bottom: $R1$ and $R2$ are considered as two distinct biological reactions when at least one input or one output differs between reactions. According to previous definitions, we generate pairs $(h1, cond1)$ and $(h2, cond2)$ for each reaction $R1$ and $R2$, where $h1$ and $h2$ are event variables and $cond1$ and $cond2$ are condition expressions for $R1$ and $R2$, respectively. Since information is transferred from A to B when $R1$ or/and $R2$ occurs, the event of the A transition is $(h1\ \mathbf{when}\ cond1)\ default\ (h2\ \mathbf{when}\ cond2)$ where $cond1 = C\ and\ (Act1\ or\ Act2)\ and\ not(Inh1\ or\ Inh2)$ and $cond2 = E\ and\ Act3\ and\ not\ Inh3$.

Supplementary Figure 2: Illustration of translation schemes for the TGF-β model.

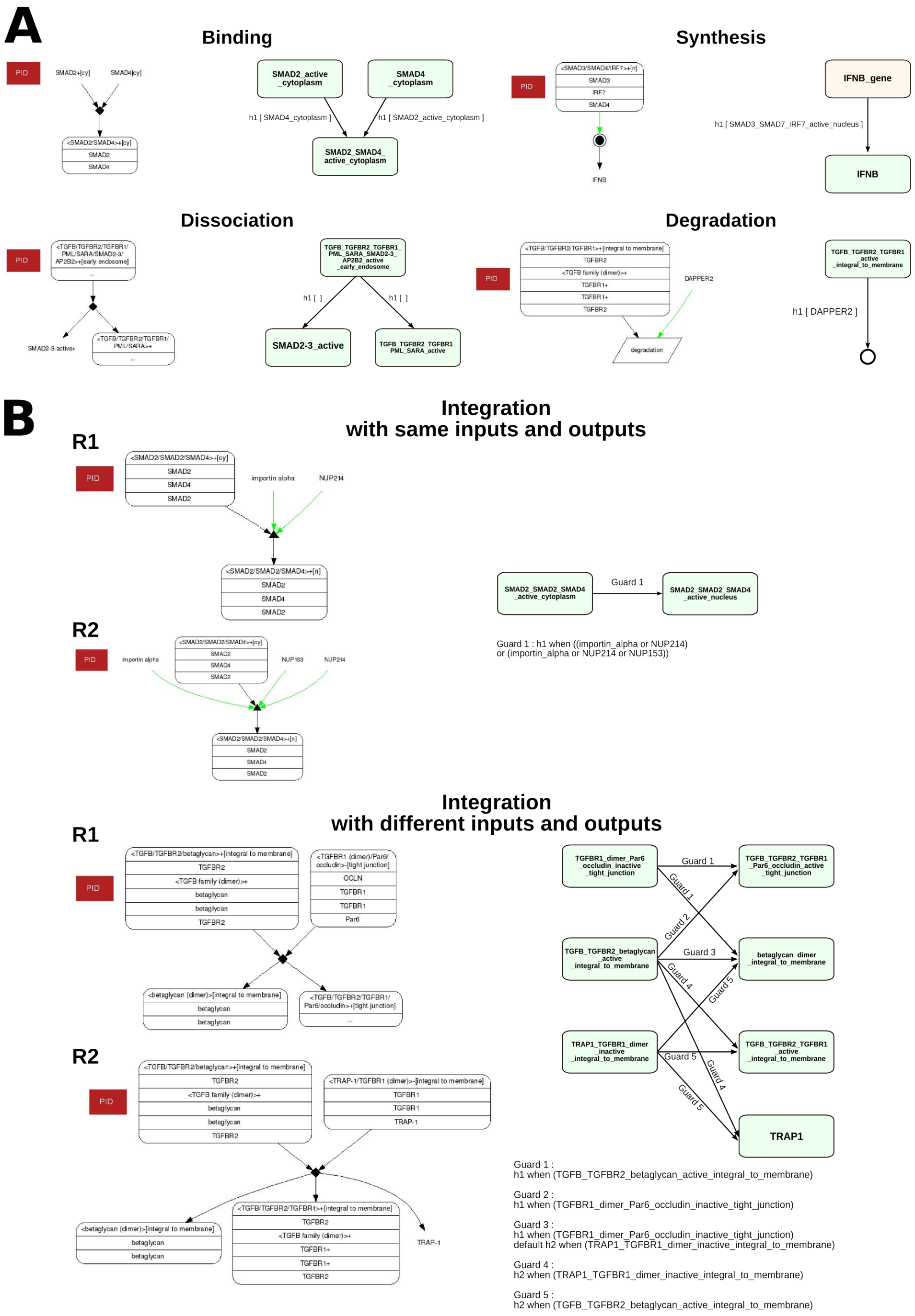

Supplementary Figure 2: Illustration of translation schemes for the TGF-β model. (**A**) Translation scheme for different categories of biological reactions from the PID database into guarded transitions. The association between SMAD2 and SMAD4 is translated into two transition from each input of the reaction to the output complex. The event is the same for both transitions since they depend on the same reaction. The dissociation of TGFB_TGFBR2_TGFBR1_PML_SARA_SMAD2-3_AP2B2_active_early_endosome complex into TGFB_TGFBR2_TGFBR1_PML_SARA and SMAD2-3 is translated into two transition with the same event. Synthesis of the TGF-β target gene IFNB is translated through a transition from a permanent place, symbolizing the gene, to the place symbolizing the protein.
The transition is conditioned by the transcriptional activator: SMAD3_SMAD7_IRF7_active_nucleus. The transition from TGFB_TGFBR2_TGFBR1_active_integral_to_membrane to a trap place (black circle) represents the degradation process activated by DAPPER2.

(**B**) Representation of the integration of reactions that share the same biomolecules. Top: the transport of SMAD2_SMAD2_SMAD4 complexes from the cytoplasm to the nucleus is described by different reactions in the PID database.
Reactions that have exactly the same input (SMAD2_SMAD2_SMAD4_active_cytoplasm) and ouput (SMAD2_SMAD2_SMAD4_active_cytoplasm) are translated into a transition with a single event. The regulators are combined using the "and" logical operator.
Bottom: different reactions have TGFB_TGFBR2_betaglycan_active_integral_to_membrane as input and betaglycan_dimer_integral_to_membrane as output, among other inputs and outputs. The guard of the transition is composed of events of reaction R1:
h1 when (TGFBR1_dimer_Par6_occludin_inactive_tight_junction) and of reaction R2:
h2 when (TRAP1_TGFBR1_dimer_inactive_integral_to_membrane).

Supplementary Figure 3: Conservation of biological annotations.

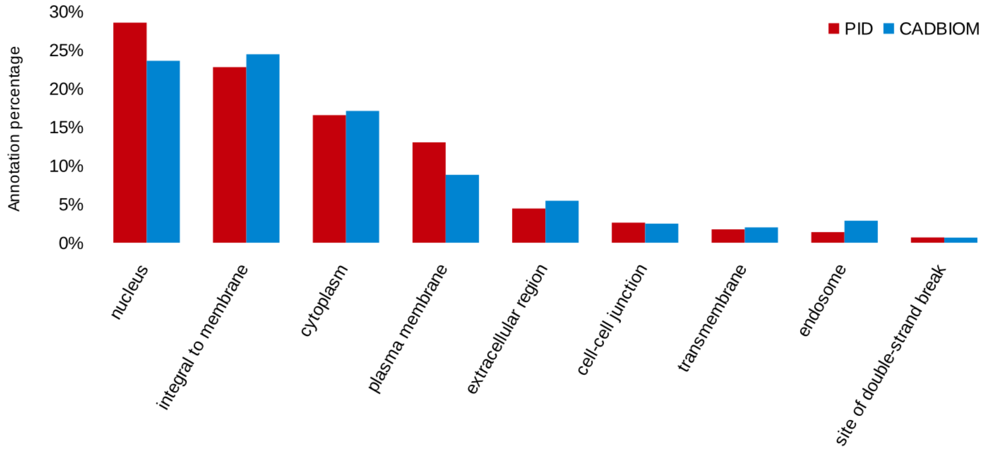

Supplementary Figure 3: Conservation of biological annotations.
The cellular component of GO ontologies of biomolecules (PID, red) and places (CADBIOM , blue) are analyzed according to their frequencies. The ten most represented location terms in PID and CADBIOM are equally distributed.

Supplementary Figure 4: Reachability of a property *P*.

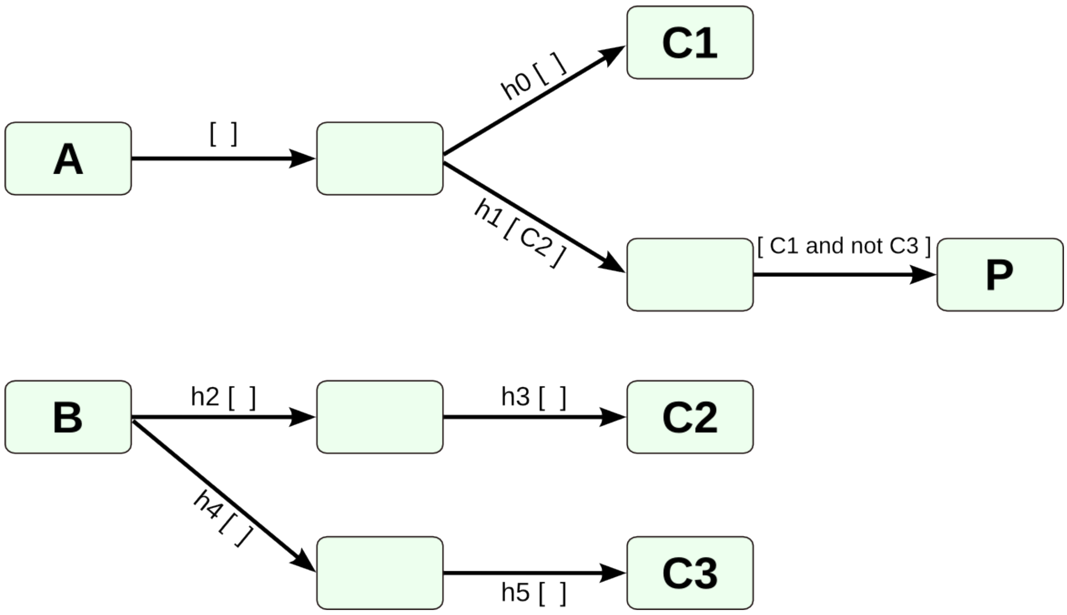

Supplementary Figure 4: Reachability of a property *P*.
When the system is initialized by activation of places A and B, the scenario $(\{A,B\},[\{h_2\},\{h_3\},\{h_0,h_1\},\{h_5\}])$ is a solution, that is, conditions that allow reaching property P in 4 steps. In this model, the scenario $(\{A,B\},[\{h_2\},\{h_3\},\{h_0,h_1\},\{\}])$ is minimal for the reachability of the place P, in 4 steps.

Supplementary Methods: mathematical semantics

Events and states

Event realization

An event is the mathematical concept that denotes a from-time-to-time occurrence. In the absence of a universal time reference, an event alone is of no interest. It requires at least another event to exist, such as an observer or a clock.

An event has a name h and a singleton domain to denote the occurrence of the event. We define $\top$ as the unique element of the event domain[1]. Occurrences of different events are equivalent as far as the domain is concerned. It is comparable to the "tick" of a clock. In general, when considering only time, we don't make any distinction between the sounds emitted by different clocks. Accordingly, it is legitimate to consider that all events have the same value.

Definition 1.1
A realization of a finite set of events $(h_i)_{i\in I}$ *is a sequence of elements of* $\{\top,\bot\}^I \setminus \{\bot^I\}$.

The sequence may be finite or infinite. The symbol $\bot$ denotes the absence of an event relative to another event. The simultaneous absence of all the events cannot occur since at least one observer must be present to mark this fact. For this reason, the multiple $\bot^I$ is excluded. The following example shows a realization of the events e_1, e_2 and e_3:

$$\begin{array}{rcccccccc} \mathbf{e}_1: & \top & \top & \bot & \bot & \top & \top & \top & \dots \\ \mathbf{e}_2: & \bot & \top & \top & \top & \bot & \bot & \top & \dots \\ \mathbf{e}_3: & \bot & \bot & \top & \bot & \bot & \bot & \top & \dots \end{array}$$

State variables

An event is well adapted to model a transient phenomenon. A new type of variable is required to model something that is persistent. This type of variable is called a **state variable**. The domain of values for state variables is any useful domain. For guarded transition systems, we will consider Boolean and finite domains.

In a realization, the value of a state variable may change. However and contrary to events, a state variable is always available. At any step or for any index of the realization, the value of a state variable is either $True$ or $False$.

The following example shows a realization of the events e_1, e_2 and the state variable A:

$$\begin{array}{rcccccccc} \mathbf{e}_1: & \top & \top & \bot & \bot & \top & \top & \top & \dots \\ \mathbf{e}_2: & \bot & \top & \top & \top & \bot & \bot & \top & \dots \\ \mathbf{A}: & F & F & F & F & T & T & T & \dots \end{array}$$

where A is a state variable and F and T stand for false and true, respectively.

This leads to the following definition:

Definition 1.2
A realization of a finite set of events and states $(h_i)_{i\in I}, (S_j)_{j\in J}$ *is a finite or infinite sequence of elements of* $(\{\top,\bot\}^I \setminus \{\bot^I\}) \times \{T,F\}^J$.

[1] $\top$ must not be confused with $True$ although it will be assimilated to this Boolean later on.

Basic operations on events and states

In many models, we need to combine events and, more generally, events and states. Operations on events are well known in computer science. The two basic operators are a merge that corresponds to multiplexing and a selection of occurrences that corresponds to under-sampling. For CADBIOM , we have borrowed the **default** operator and the **when** operator from the SIGNAL language with semantics close to SIGNAL semantics.

The default operator

The **default** operator merges the two events h_1 and h_2 or, more precisely, the occurrences of the events in any realization. In the following example, we assume that there are more events. This legitimizes the case where h_1 and h_2 are simultaneously absent.

$$\begin{array}{rcccccccc} \mathbf{h}_1: & \top & \top & \bot & \bot & \top & \bot & \top & \dots \\ \mathbf{h}_2: & \bot & \top & \top & \top & \bot & \bot & \top & \dots \\ \mathbf{h}_1 \textbf{ default } \mathbf{h}_2: & \top & \top & \top & \top & \top & \bot & \top & \dots \end{array}$$

This operator on events is commutative.

For the mathematically inclined reader, we provide a rigorous definition. We first define an operator $\uparrow$ on $\{\top, \bot\}$ by following the rules:

$$\begin{array}{rcl} \top \uparrow \top & = & \top \\ \top \uparrow \bot & = & \top \\ \bot \uparrow \top & = & \top \\ \bot \uparrow \bot & = & \bot \end{array}$$

We then define **default** by its semantics:

Definition 1.3
Given two events h_1 and h_2, $h = h_1$ **default** *h_2 if and only if, for any realizations s of (h_1, h_2, h), the relation $s_i^h = s1_i^{h_1} \uparrow s2_i^{h_2}$ is satisfied.*

The when operator

The **when** operator is an operator between events and logical combinations of state variables. Since state variables have a Boolean domain, it is possible to write propositional logic formulas with state variables. At each instant of a realization, the formula can be evaluated since state variables always have values.

The **when** operator selects occurrences of an event when the propositional formula evaluates to *True* on its right hand side. For example:

$$\begin{array}{rcccccccc} \mathbf{h}_1: & \top & \top & \bot & \bot & \top & \top & \top & \dots \\ \mathbf{B}: & F & T & F & T & F & T & F & \dots \\ \mathbf{h}_1 \textbf{ when } \mathbf{B}: & \bot & \top & \bot & \bot & \bot & \top & \bot & \dots \end{array}$$

where B is a state variable.

For a more mathematical definition, we need to introduce the operator $\downarrow$ with the rules:

$$\begin{array}{rcl} \top \downarrow True & = & \top \\ \top \downarrow False & = & \bot \\ \bot \downarrow True & = & \bot \\ \bot \downarrow False & = & \bot \end{array}$$

We then define **when** by its semantics:

Definition 1.4
*Given an event h_1 and a state propositional formula $B(X)$ where X represents a multiple of state variables, $h = h_1$ **when** $B(X)$ if and only if for all realizations s of (h_1, X, h), the relation $s_i^h = s1_i^{h_1} \downarrow B(x_i)$ is satisfied.*

The logical operators $\vee$, $\wedge$ and $\neg$ are implemented in CADBIOM . They can be used in the condition part of a transition guard with place names as state variable names. The **default** and **when** operators are also implemented and can be used in the event of a transition guard. The right hand side operand of a **when** must be a propositional formula with state variables (and $True$ and $False$ constants).

Extension to signals

The event concept naturally generalizes to the signal concept. Signals are useful to define the mathematical semantics of guarded transition-based models. With the extension of events to signals, it becomes possible to write the evolution equation of the dynamic system into relatively simple mathematical formulas.

A signal is a generalization of an event. Contrary to an event, a signal can have an arbitrary domain of values. An event is simply a signal with $\{\top\}$ as domain. Given a family $(Z_i)_{i\in I}$ of signals with domains $(D_i)_{i\in I}$, a realization is a sequence of multiples $(z_i^n)_{i\in I}$ such that $z_i^n \in D_i \cup \{\perp\}$. Again, the multiple $\perp^I$ is excluded.

Extending the **default** operator to signals, we face a new problem. If, in a realization of Z_1 and Z_2, we have for some instant $z_1 \neq \perp$ and $z_2 \neq \perp$, we have to decide which value to choose. Here, we decided to keep the value of the left hand side operand. Note that, in general, the domain of a signal built with the default operator is the union of the domains of the operands. In most cases, **default** is used only with operand signals that have the same domain. We will only consider Boolean domains.

The domain of an event is an arbitrary singleton. To combine events and Boolean signals in a mathematical formula, we will identify the $\top$ value with $True$. With this identification, an event is assimilated to a Boolean signal with constant value $True$. It is also possible to extend Boolean operations to Boolean signals with different semantics. Since general operations are not required on Boolean signals, they will not be discussed and only the negation of an event which is assimilated to a Boolean signal with the constant value $False$ need be considered.

A state variable can be assimilated to a signal. In any realization and at any instant, the value associated with a state variable is different from $\perp$. A state is present in any realization, it acts as memory in a computer. A state, an event and the negation of an event are limit cases of signals. They are either always present (state) or always have the same value (event or event negation). This special feature is essential to obtain a simple encoding of guarded-transition model dynamics into logical clauses.

Semantic of guarded transitions

The firing of a transition is an event. Given a guarded transition $A \xrightarrow{h[C]} B$, we define a new event, called the transition event, as:

$$h_{tr} = h \textbf{ when } (A \wedge C)$$

The transition event h_{tr} is present if and only if the three conditions for transition firing are satisfied.

If we focus on the evolution of the source and target places when a transition is fired, informal semantics state that:

- the source is inactivated
- the target is activated

The evolution function of a state B relies on the current value B_k at step k to the next value B_{k+1} at step $k+1$. Traditionally the current value is denoted as B and the next value B'. With this notation, the evolution of the target, upon possible firing of one transition, is described by:

$$B' = h_{tr} \textbf{ default } B$$

which must be interpreted with the extension of the operator **default** to signals. The state variables are considered as signals and the events are considered as signals with the value $True$. The priority of the left operand of the **default** operator is essential for defining correct semantics. When the transition is fired (h_{tr} is present), the state takes the value of the **default** left hand side operator ($True$) regardless of the preceding value. When it isn't fired, the value of the state remains unchanged.

Using the same conventional notations, the evolution of the source is formalized by:

$$A' = \neg h_{tr} \textbf{ default } A$$

reflecting the inactivation of the source when the transition is fired. Again, we use the extensions introduced at the end of the preceding section.

In general, several transitions are adjacent to a place. For a place, we call an in-transition a transition having the place as target. The set of in-transitions of a place A is denoted $T_{in}(A)$. A transition having the place as source will be called an out-transition and the set of out-transitions of a place A is denoted $T_{out}(A)$. When there is no ambiguity, the name of the place is omitted.

The state of a place changes if either an in-transition or an out-transition is fired. In case of simultaneous firing we apply the rule:

- activation prevails overs inactivation.

We define two events associated with a place A by:

$$\begin{array}{rcl} h_{in} & = & \textbf{default}_{\,t_r \in T_{in}} h_{tr} \\ h_{out} & = & \textbf{default}_{\,t_r \in T_{out}} h_{tr} \end{array}$$

The h_{in} event is present if at least one of the in-transitions is fired. The h_{out} event is present if at least one of the out-transitions is fired. The mathematical formalization of the rules is given by:

$$A' = (h_{in} \textbf{ default } \neg h_{out}) \textbf{ default } A$$

The semantics of **default** on signals are again essential to obtain a correct formalization of the priority rule.

Guarded transition systems

With the rigorous definition of the semantics of a transition, it is straightforward to verify that the two guarded transitions $A \stackrel{h[C]}{\rightarrow} B$ and $A \stackrel{(h \textbf{when } C)[]}{\longrightarrow} B$ are semantically equivalent. They induce the same dynamics on the state variables. Taking advantage of this, we will consider guarded transitions without condition.

Definition 1.5
A guarded transition system (GTS) is a triplet $(\mathscr{P}, \mathscr{H}, \mathscr{T})$ *where:*

- $\mathscr{P}$ *is a finite set of places*
- $\mathscr{H}$ *is a finite set of free events*
- $\mathscr{T}$ *is a finite set of triplets* (A,B,h) *where* $A,B \in \mathscr{P}$ *and h is an event built on* $\mathscr{P}$ *and* $\mathscr{H}$

The state of a guard transition system is a Boolean multiple in $\{T,F\}^{|\mathscr{P}|}$. This state is equivalently described by the set $\mathscr{A}$ of activated places. The evolution of the state depends also on the events H present at the current evolution step. This motivates the following definition:

Definition 1.6
A configuration of a guarded transition system $(\mathscr{P}, \mathscr{H}, \mathscr{T})$ *is a couple* $(\mathscr{A}, H)$ *where* $\mathscr{A}$ *is a subset of* $\mathscr{P}$ *representing activated places, and* H *is a subset of free events that are present at the evolution step.*

Property search

Because of the semantics of guarded transitions, the evolution of a guarded transition system is completely determined by the initial activated places and a sequence of free events.

Definition 1.7
A scenario is a couple $(\mathscr{A}_0, (\mathscr{E}_0, \mathscr{E}_1, \ldots, \mathscr{E}_{n-1}))$ *where*

- $\mathscr{A}_0$ *is the set of activated places at initialization*
- $(\mathscr{E}_0, \mathscr{E}_1, \ldots, \mathscr{E}_{n-1})$ *is a sequence of subset of free events.*

n *is the length or the horizon of the scenario.*

A sequence of configurations $(\mathscr{A}_k, \mathscr{E}_k)$ is associated with a scenario where $\mathscr{A}_k$ is the set of activated places after the kth step. $\mathscr{A}_n$ is the set of activated places reached by the scenario. A state property represented by a logical formula f on places is reached by the scenario if for some $k \leq n$, $\mathscr{A}_k$ is a model of the formula f.

From dynamic model to logical formula
To perform analysis satisfactorily, we first translate the dynamic systems into propositional logic. The value of each place depends on its surrounding transitions as follows:

$$I^i \overset{G^i}{\rightarrow} x \overset{G^j}{\rightarrow} O^j$$

where I^i the input place of an incoming transition to x with the guard G^i, O^j is the output place of an outgoing transition from x with the guard G^j. The value of place x at step 1 can be written as:

$$x_1 \Leftrightarrow \Big[\bigvee_{i \in [0,m]} (I_0^i \wedge G_0^i) \Big] \vee \Big[x_0 \wedge \neg\, (x_0 \wedge \bigvee_{j \in [0,l]} (G_0^j) \Big]$$

where m and l are the numbers of incoming and outgoing transitions. This formula is translated into conjunction normal form using the Tseitin translation.

Unfolding the trajectory
The evolution rule is summarized as $X_n = f(X_{n-1})$, where Xn is the set of place values at step n. This formula first describes the evolution between steps 0 and 1. We create new literals to unfold the trajectory to the step n as follows: $X_1 = f(X_0)$; $X_2 = f(X_1); \ldots;$ $X_n = f(X_{n-1})$. The final formula is next given to the SAT solver that allocates the value for each litterals to verify the formula.
